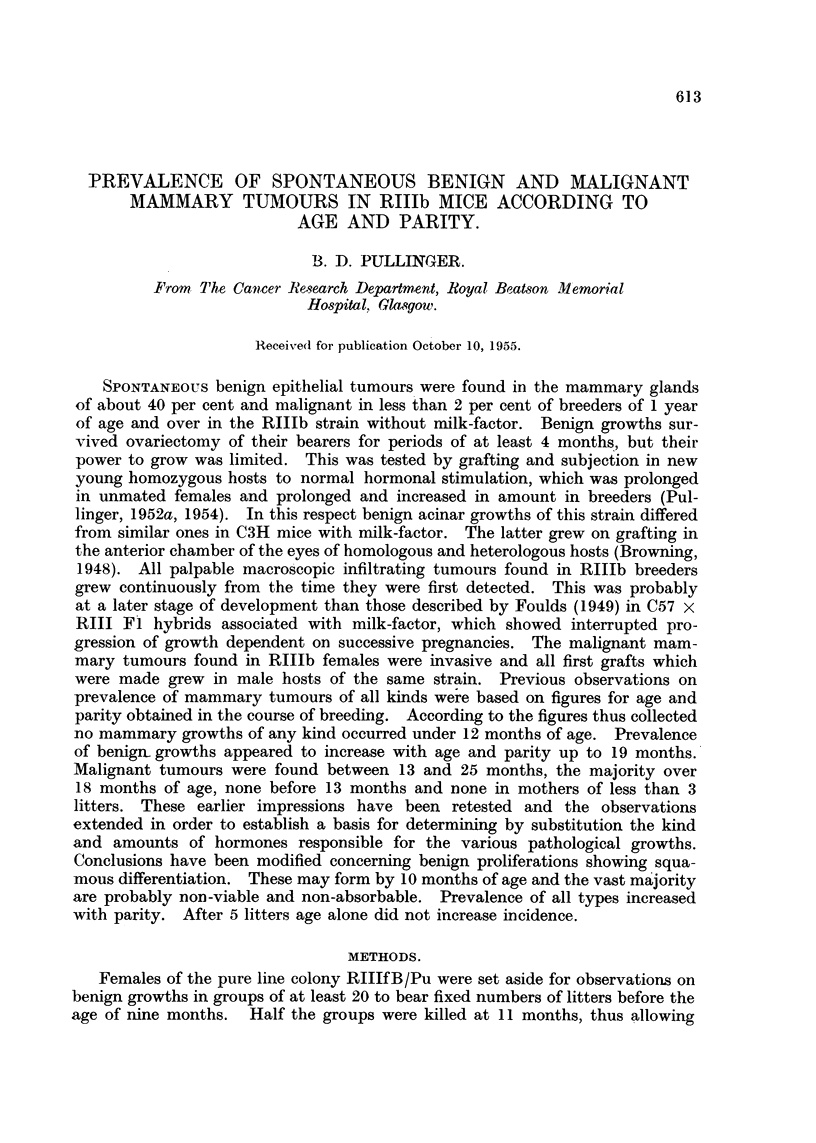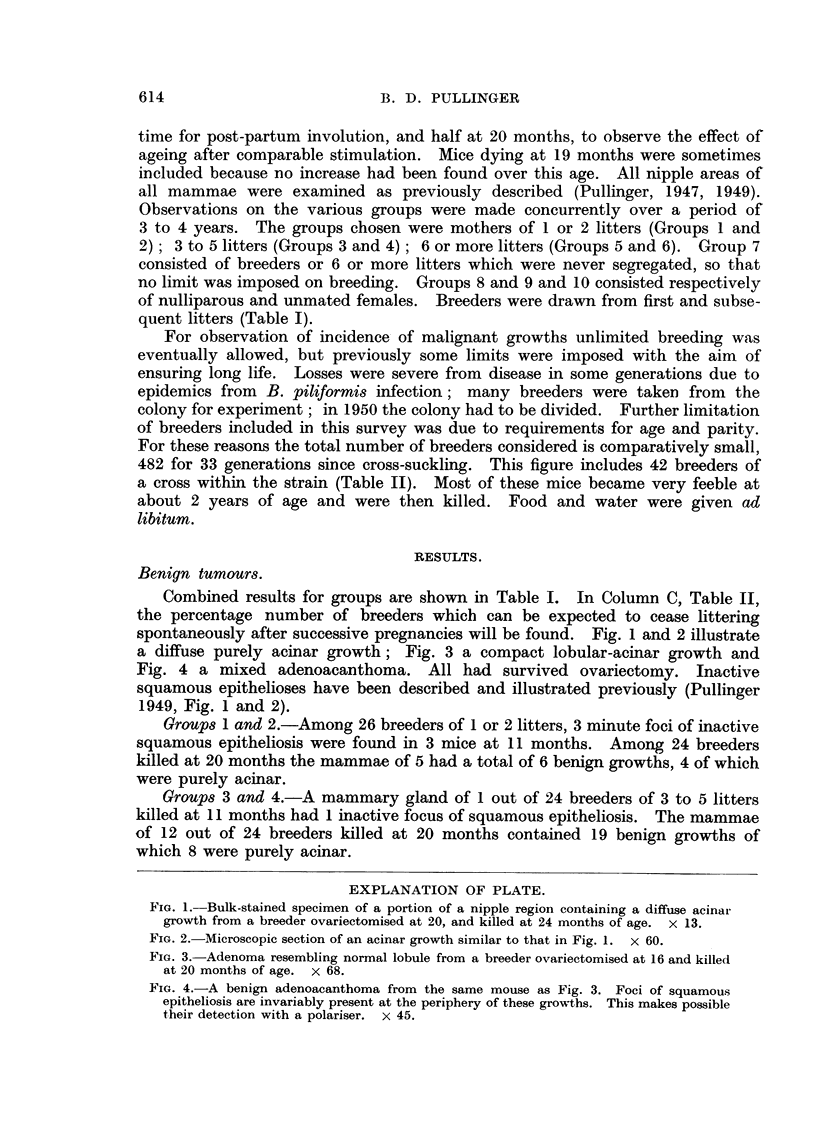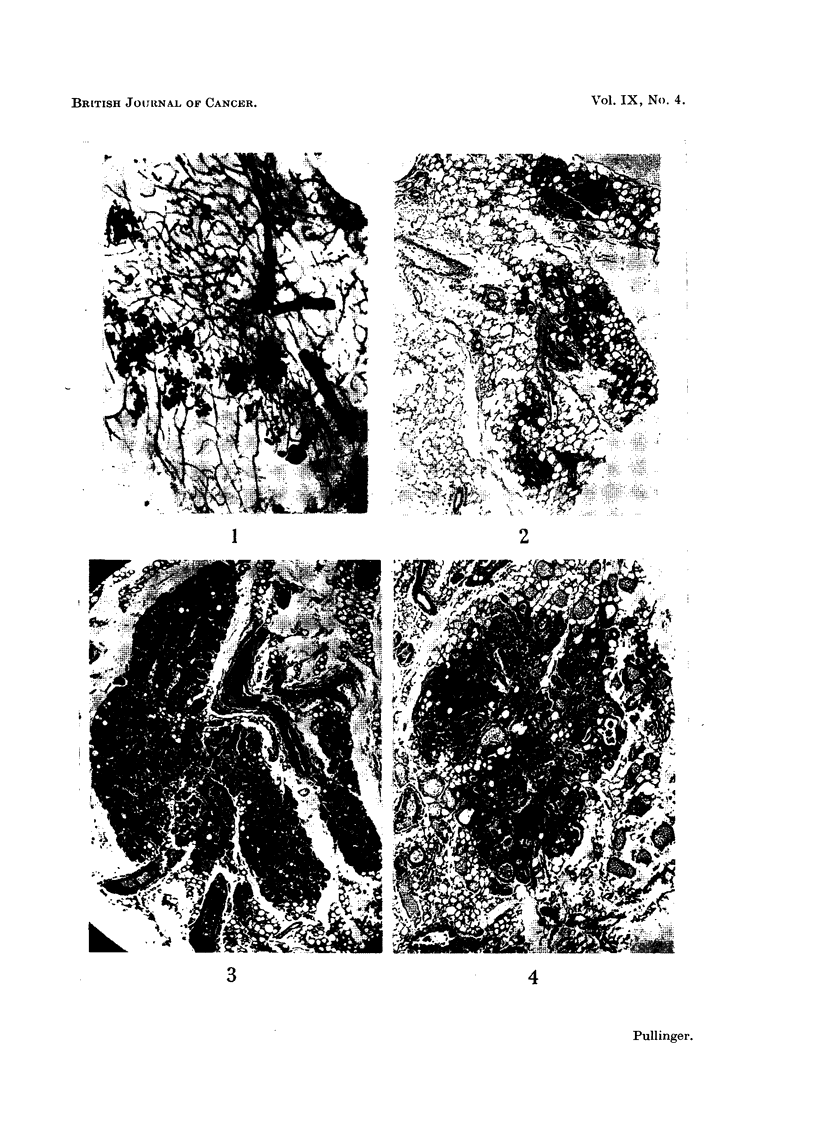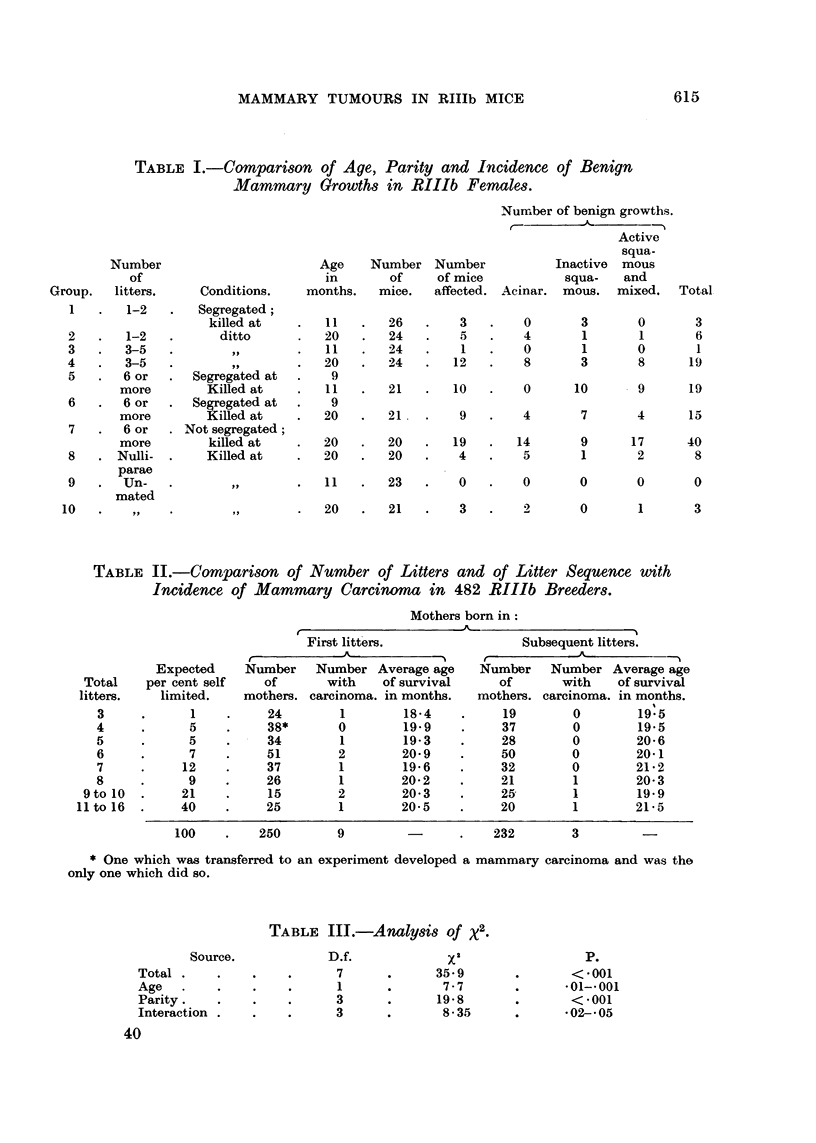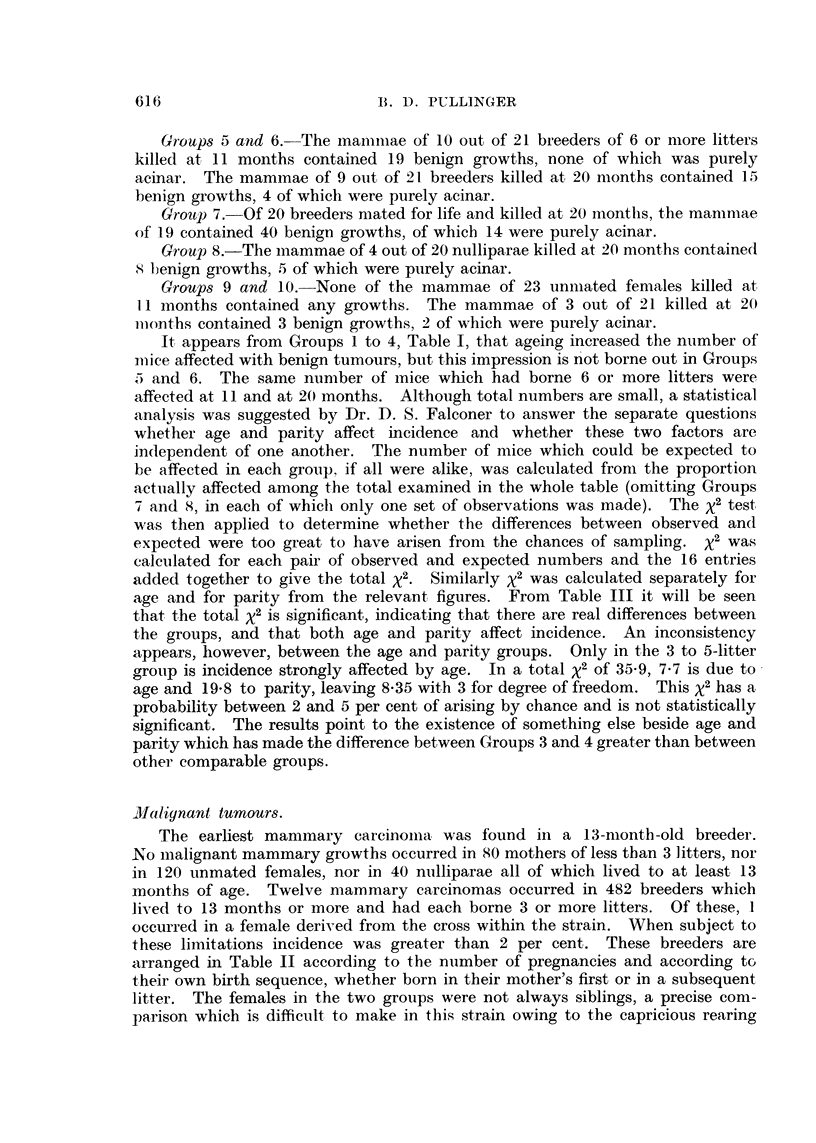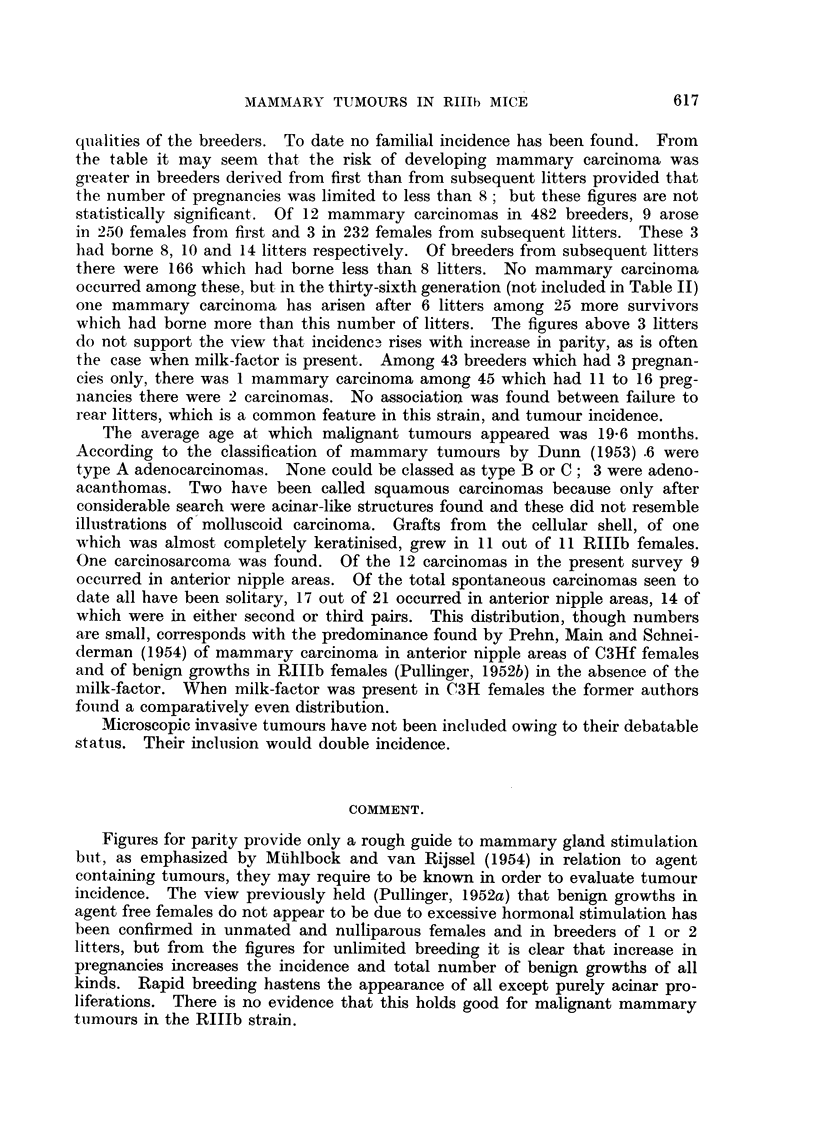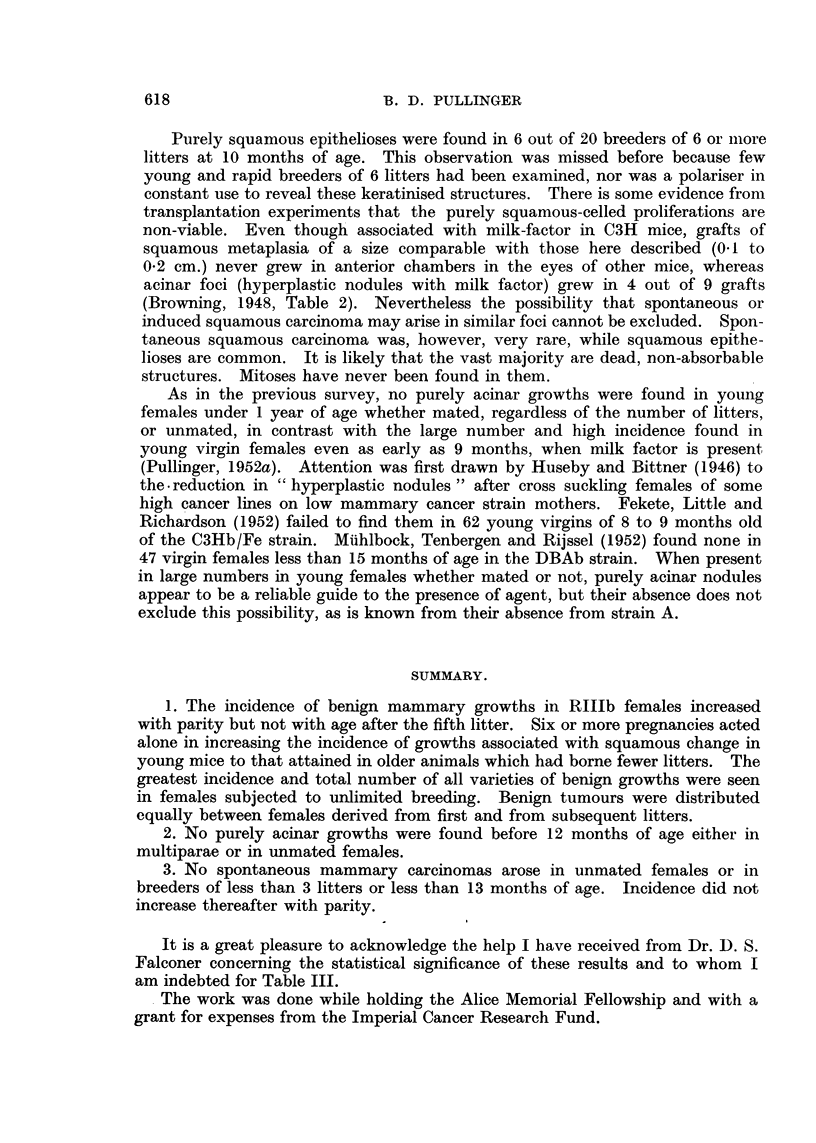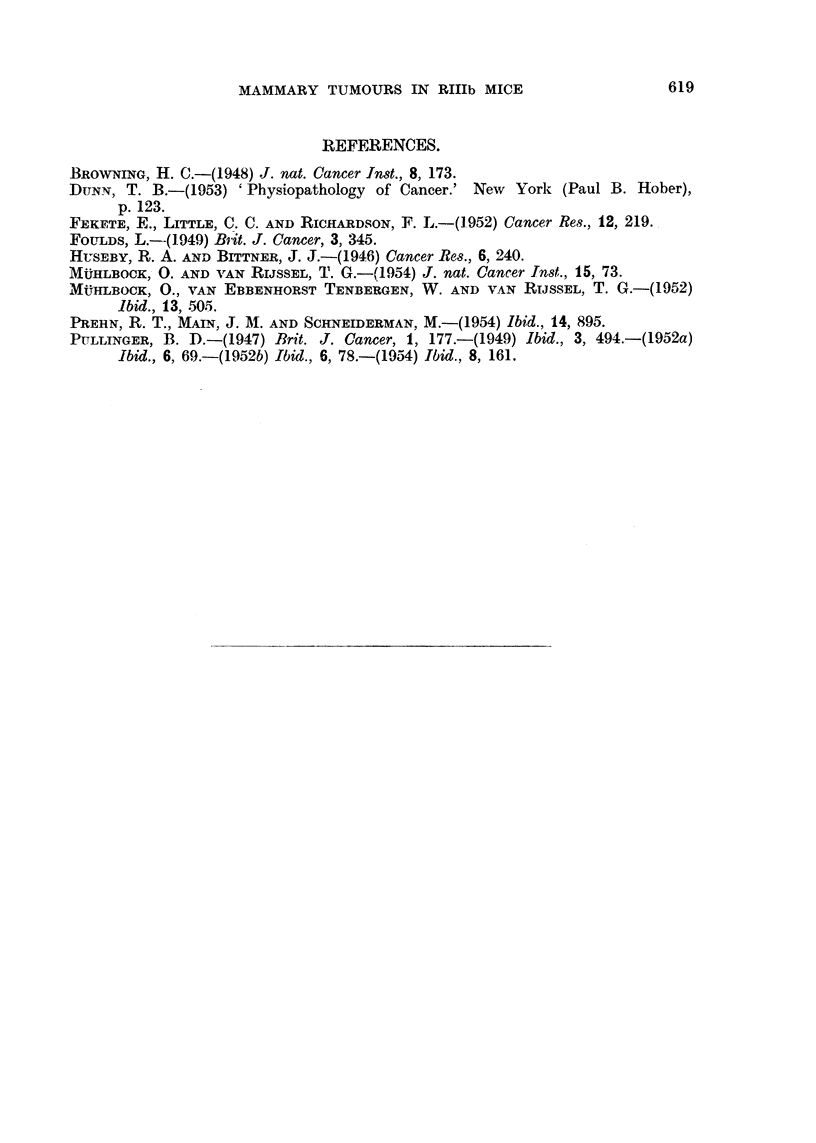# Prevalence of Spontaneous Benign and Malignant Mammary Tumours in RIIIb Mice According to Age and Parity

**DOI:** 10.1038/bjc.1955.64

**Published:** 1955-12

**Authors:** B. D. Pullinger

## Abstract

**Images:**


### PREVALENCE OF SPONTANEOUS BENIGN AND MALIGNANT MAMMARY TUMOURS IN RIIIb MICE ACCORDING TO AGE AND PARITY.

B. D. PULLINGER.

*From The Cancer Research Department, Royal Beatson Memorial Hospital, Glasgow.*

Received for publication October 10, 1955.

SPONTANEOUS benign epithelial tumours were found in the mammary glands of about 40 per cent and malignant in less than 2 per cent of breeders of 1 year of age and over in the RIIIb strain without milk-factor. Benign growths survived ovariectomy of their bearers for periods of at least 4 months, but their power to grow was limited. This was tested by grafting and subjection in new young homozygous hosts to normal hormonal stimulation, which was prolonged in unmated females and prolonged and increased in amount in breeders (Pullinger, 1952*a*, 1954). In this respect benign acinar growths of this strain differed from similar ones in C3H mice with milk-factor. The latter grew on grafting in the anterior chamber of the eyes of homologous and heterologous hosts (Browning, 1948). All palpable macroscopic infiltrating tumours found in RIIIb breeders grew continuously from the time they were first detected. This was probably at a later stage of development than those described by Foulds (1949) in C57 × RIII F1 hybrids associated with milk-factor, which showed interrupted progression of growth dependent on successive pregnancies. The malignant mammary tumours found in RIIIb females were invasive and all first grafts which were made grew in male hosts of the same strain. Previous observations on prevalence of mammary tumours of all kinds were based on figures for age and parity obtained in the course of breeding. According to the figures thus collected no mammary growths of any kind occurred under 12 months of age. Prevalence of benign growths appeared to increase with age and parity up to 19 months. Malignant tumours were found between 13 and 25 months, the majority over 18 months of age, none before 13 months and none in mothers of less than 3 litters. These earlier impressions have been retested and the observations extended in order to establish a basis for determining by substitution the kind and amounts of hormones responsible for the various pathological growths. Conclusions have been modified concerning benign proliferations showing squamous differentiation. These may form by 10 months of age and the vast majority are probably non-viable and non-absorbable. Prevalence of all types increased with parity. After 5 litters age alone did not increase incidence.

### METHODS.

Females of the pure line colony RIIIfB/Pu were set aside for observations on benign growths in groups of at least 20 to bear fixed numbers of litters before the age of nine months. Half the groups were killed at 11 months, thus allowing time for post-partum involution, and half at 20 months, to observe the effect of ageing after comparable stimulation. Mice dying at 19 months were sometimes included because no increase had been found over this age. All nipple areas of all mammae were examined as previously described (Pullinger, 1947, 1949). Observations on the various groups were made concurrently over a period of 3 to 4 years. The groups chosen were mothers of 1 or 2 litters (Groups 1 and 2); 3 to 5 litters (Groups 3 and 4); 6 or more litters (Groups 5 and 6). Group 7 consisted of breeders or 6 or more litters which were never segregated, so that no limit was imposed on breeding. Groups 8 and 9 and 10 consisted respectively of nulliparous and unmated females. Breeders were drawn from first and subsequent litters (Table I).

For observation of incidence of malignant growths unlimited breeding was eventually allowed, but previously some limits were imposed with the aim of ensuring long life. Losses were severe from disease in some generations due to epidemics from *B. piliformis* infection; many breeders were taken from the colony for experiment; in 1950 the colony had to be divided. Further limitation of breeders included in this survey was due to requirements for age and parity. For these reasons the total number of breeders considered is comparatively small, 482 for 33 generations since cross-suckling. This figure includes 42 breeders of a cross within the strain (Table II). Most of these mice became very feeble at about 2 years of age and were then killed. Food and water were given *ad libitum.*

### RESULTS.

### *Benign tumours.*

Combined results for groups are shown in Table I. In Column C, Table II, the percentage number of breeders which can be expected to cease littering spontaneously after successive pregnancies will be found. Fig. 1 and 2 illustrate a diffuse purely acinar growth; Fig. 3 a compact lobular-acinar growth and Fig. 4 a mixed adenoacanthoma. All had survived ovariectomy. Inactive squamous epithelioses have been described and illustrated previously (Pullinger 1949, Fig. 1 and 2).

*Groups* 1 *and* 2.—Among 26 breeders of 1 or 2 litters, 3 minute foci of inactive squamous epitheliosis were found in 3 mice at 11 months. Among 24 breeders killed at 20 months the mammae of 5 had a total of 6 benign growths, 4 of which were purely acinar.

*Groups* 3 *and* 4.—A mammary gland of 1 out of 24 breeders of 3 to 5 litters killed at 11 months had 1 inactive focus of squamous epitheliosis. The mammae of 12 out of 24 breeders killed at 20 months contained 19 benign growths of which 8 were purely acinar.

---

EXPLANATION OF PLATE.

FIG. 1.—Bulk-stained specimen of a portion of a nipple region containing a diffuse acinar growth from a breeder ovariectomised at 20, and killed at 24 months of age. × 13.

FIG. 2.—Microscopic section of an acinar growth similar to that in Fig. 1. × 60.

FIG. 3.—Adenoma resembling normal lobule from a breeder ovariectomised at 16 and killed at 20 months of age. × 68.

FIG. 4.—A benign adenoacanthoma from the same mouse as Fig. 3. Foci of squamous epitheliosis are invariably present at the periphery of these growths. This makes possible their detection with a polariser. × 45.

1

2

3

4

Pullinger.

TABLE I.—*Comparison of Age, Parity and Incidence of Benign Mammary Growths in RIIIb Females.*

| Group. | Number of litters. | Conditions. | Age in months. | Number of mice. | Number of mice affected. | Number of benign growths. Acinar. | Inactive squamous. | Active squamous and mixed. | Total |
|---|---|---|---|---|---|---|---|---|---|
| 1 | 1–2 | Segregated; killed at | 11 | 26 | 3 | 0 | 3 | 0 | 3 |
| 2 | 1–2 | ditto | 20 | 24 | 5 | 4 | 1 | 1 | 6 |
| 3 | 3–5 | ,, | 11 | 24 | 1 | 0 | 1 | 0 | 1 |
| 4 | 3–5 | ,, | 20 | 24 | 12 | 8 | 3 | 8 | 19 |
| 5 | 6 or more | Segregated at<br>Killed at | 9<br>11 | 21 | 10 | 0 | 10 | 9 | 19 |
| 6 | 6 or more | Segregated at<br>Killed at | 9<br>20 | 21 | 9 | 4 | 7 | 4 | 15 |
| 7 | 6 or more | Not segregated; killed at | 20 | 20 | 19 | 14 | 9 | 17 | 40 |
| 8 | Nulliparae | Killed at | 20 | 20 | 4 | 5 | 1 | 2 | 8 |
| 9 | Unmated | ,, | 11 | 23 | 0 | 0 | 0 | 0 | 0 |
| 10 | ,, | ,, | 20 | 21 | 3 | 2 | 0 | 1 | 3 |

TABLE II.—*Comparison of Number of Litters and of Litter Sequence with Incidence of Mammary Carcinoma in* 482 *RIIIb Breeders.*

| | | Mothers born in: First litters. | | | Subsequent litters. | | |
|---|---|---|---|---|---|---|---|
| Total litters. | Expected per cent self limited. | Number of mothers. | Number with carcinoma. | Average age of survival in months. | Number of mothers. | Number with carcinoma. | Average age of survival in months. |
| 3 | 1 | 24 | 1 | 18·4 | 19 | 0 | 19·5 |
| 4 | 5 | 38* | 0 | 19·9 | 37 | 0 | 19·5 |
| 5 | 5 | 34 | 1 | 19·3 | 28 | 0 | 20·6 |
| 6 | 7 | 51 | 2 | 20·9 | 50 | 0 | 20·1 |
| 7 | 12 | 37 | 1 | 19·6 | 32 | 0 | 21·2 |
| 8 | 9 | 26 | 1 | 20·2 | 21 | 1 | 20·3 |
| 9 to 10 | 21 | 15 | 2 | 20·3 | 25 | 1 | 19·9 |
| 11 to 16 | 40 | 25 | 1 | 20·5 | 20 | 1 | 21·5 |
| | 100 | 250 | 9 | — | 232 | 3 | — |

* One which was transferred to an experiment developed a mammary carcinoma and was the only one which did so.

TABLE III.—*Analysis of* $\chi^2$.

| Source. | D.f. | $\chi^2$ | P. |
|---|---|---|---|
| Total | 7 | 35·9 | <·001 |
| Age | 1 | 7·7 | ·01–·001 |
| Parity | 3 | 19·8 | <·001 |
| Interaction | 3 | 8·35 | ·02–·05 |

*Groups* 5 *and* 6.—The mammae of 10 out of 21 breeders of 6 or more litters killed at 11 months contained 19 benign growths, none of which was purely acinar. The mammae of 9 out of 21 breeders killed at 20 months contained 15 benign growths, 4 of which were purely acinar.

*Group* 7.—Of 20 breeders mated for life and killed at 20 months, the mammae of 19 contained 40 benign growths, of which 14 were purely acinar.

*Group* 8.—The mammae of 4 out of 20 nulliparae killed at 20 months contained 8 benign growths, 5 of which were purely acinar.

*Groups* 9 *and* 10.—None of the mammae of 23 unmated females killed at 11 months contained any growths. The mammae of 3 out of 21 killed at 20 months contained 3 benign growths, 2 of which were purely acinar.

It appears from Groups 1 to 4, Table I, that ageing increased the number of mice affected with benign tumours, but this impression is not borne out in Groups 5 and 6. The same number of mice which had borne 6 or more litters were affected at 11 and at 20 months. Although total numbers are small, a statistical analysis was suggested by Dr. D. S. Falconer to answer the separate questions whether age and parity affect incidence and whether these two factors are independent of one another. The number of mice which could be expected to be affected in each group, if all were alike, was calculated from the proportion actually affected among the total examined in the whole table (omitting Groups 7 and 8, in each of which only one set of observations was made). The $\chi^2$ test was then applied to determine whether the differences between observed and expected were too great to have arisen from the chances of sampling. $\chi^2$ was calculated for each pair of observed and expected numbers and the 16 entries added together to give the total $\chi^2$. Similarly $\chi^2$ was calculated separately for age and for parity from the relevant figures. From Table III it will be seen that the total $\chi^2$ is significant, indicating that there are real differences between the groups, and that both age and parity affect incidence. An inconsistency appears, however, between the age and parity groups. Only in the 3 to 5-litter group is incidence strongly affected by age. In a total $\chi^2$ of 35·9, 7·7 is due to age and 19·8 to parity, leaving 8·35 with 3 for degree of freedom. This $\chi^2$ has a probability between 2 and 5 per cent of arising by chance and is not statistically significant. The results point to the existence of something else beside age and parity which has made the difference between Groups 3 and 4 greater than between other comparable groups.

*Malignant tumours.*

The earliest mammary carcinoma was found in a 13-month-old breeder. No malignant mammary growths occurred in 80 mothers of less than 3 litters, nor in 120 unmated females, nor in 40 nulliparae all of which lived to at least 13 months of age. Twelve mammary carcinomas occurred in 482 breeders which lived to 13 months or more and had each borne 3 or more litters. Of these, 1 occurred in a female derived from the cross within the strain. When subject to these limitations incidence was greater than 2 per cent. These breeders are arranged in Table II according to the number of pregnancies and according to their own birth sequence, whether born in their mother's first or in a subsequent litter. The females in the two groups were not always siblings, a precise comparison which is difficult to make in this strain owing to the capricious rearing qualities of the breeders. To date no familial incidence has been found. From the table it may seem that the risk of developing mammary carcinoma was greater in breeders derived from first than from subsequent litters provided that the number of pregnancies was limited to less than 8 ; but these figures are not statistically significant. Of 12 mammary carcinomas in 482 breeders, 9 arose in 250 females from first and 3 in 232 females from subsequent litters. These 3 had borne 8, 10 and 14 litters respectively. Of breeders from subsequent litters there were 166 which had borne less than 8 litters. No mammary carcinoma occurred among these, but in the thirty-sixth generation (not included in Table II) one mammary carcinoma has arisen after 6 litters among 25 more survivors which had borne more than this number of litters. The figures above 3 litters do not support the view that incidence rises with increase in parity, as is often the case when milk-factor is present. Among 43 breeders which had 3 pregnancies only, there was 1 mammary carcinoma among 45 which had 11 to 16 pregnancies there were 2 carcinomas. No association was found between failure to rear litters, which is a common feature in this strain, and tumour incidence.

The average age at which malignant tumours appeared was 19·6 months. According to the classification of mammary tumours by Dunn (1953) 6 were type A adenocarcinomas. None could be classed as type B or C ; 3 were adenoacanthomas. Two have been called squamous carcinomas because only after considerable search were acinar-like structures found and these did not resemble illustrations of molluscoid carcinoma. Grafts from the cellular shell, of one which was almost completely keratinised, grew in 11 out of 11 RIIIb females. One carcinosarcoma was found. Of the 12 carcinomas in the present survey 9 occurred in anterior nipple areas. Of the total spontaneous carcinomas seen to date all have been solitary, 17 out of 21 occurred in anterior nipple areas, 14 of which were in either second or third pairs. This distribution, though numbers are small, corresponds with the predominance found by Prehn, Main and Schneiderman (1954) of mammary carcinoma in anterior nipple areas of C3Hf females and of benign growths in RIIIb females (Pullinger, 1952*b*) in the absence of the milk-factor. When milk-factor was present in C3H females the former authors found a comparatively even distribution.

Microscopic invasive tumours have not been included owing to their debatable status. Their inclusion would double incidence.

### COMMENT.

Figures for parity provide only a rough guide to mammary gland stimulation but, as emphasized by Mühlbock and van Rijssel (1954) in relation to agent containing tumours, they may require to be known in order to evaluate tumour incidence. The view previously held (Pullinger, 1952*a*) that benign growths in agent free females do not appear to be due to excessive hormonal stimulation has been confirmed in unmated and nulliparous females and in breeders of 1 or 2 litters, but from the figures for unlimited breeding it is clear that increase in pregnancies increases the incidence and total number of benign growths of all kinds. Rapid breeding hastens the appearance of all except purely acinar proliferations. There is no evidence that this holds good for malignant mammary tumours in the RIIIb strain.

Purely squamous epithelioses were found in 6 out of 20 breeders of 6 or more litters at 10 months of age. This observation was missed before because few young and rapid breeders of 6 litters had been examined, nor was a polariser in constant use to reveal these keratinised structures. There is some evidence from transplantation experiments that the purely squamous-celled proliferations are non-viable. Even though associated with milk-factor in C3H mice, grafts of squamous metaplasia of a size comparable with those here described (0·1 to 0·2 cm.) never grew in anterior chambers in the eyes of other mice, whereas acinar foci (hyperplastic nodules with milk factor) grew in 4 out of 9 grafts (Browning, 1948, Table 2). Nevertheless the possibility that spontaneous or induced squamous carcinoma may arise in similar foci cannot be excluded. Spontaneous squamous carcinoma was, however, very rare, while squamous epithelioses are common. It is likely that the vast majority are dead, non-absorbable structures. Mitoses have never been found in them.

As in the previous survey, no purely acinar growths were found in young females under 1 year of age whether mated, regardless of the number of litters, or unmated, in contrast with the large number and high incidence found in young virgin females even as early as 9 months, when milk factor is present (Pullinger, 1952*a*). Attention was first drawn by Huseby and Bittner (1946) to the reduction in " hyperplastic nodules " after cross suckling females of some high cancer lines on low mammary cancer strain mothers. Fekete, Little and Richardson (1952) failed to find them in 62 young virgins of 8 to 9 months old of the C3Hb/Fe strain. Mühlbock, Tenbergen and Rijssel (1952) found none in 47 virgin females less than 15 months of age in the DBAb strain. When present in large numbers in young females whether mated or not, purely acinar nodules appear to be a reliable guide to the presence of agent, but their absence does not exclude this possibility, as is known from their absence from strain A.

### SUMMARY.

1. The incidence of benign mammary growths in RIIIb females increased with parity but not with age after the fifth litter. Six or more pregnancies acted alone in increasing the incidence of growths associated with squamous change in young mice to that attained in older animals which had borne fewer litters. The greatest incidence and total number of all varieties of benign growths were seen in females subjected to unlimited breeding. Benign tumours were distributed equally between females derived from first and from subsequent litters.

2. No purely acinar growths were found before 12 months of age either in multiparae or in unmated females.

3. No spontaneous mammary carcinomas arose in unmated females or in breeders of less than 3 litters or less than 13 months of age. Incidence did not increase thereafter with parity.

It is a great pleasure to acknowledge the help I have received from Dr. D. S. Falconer concerning the statistical significance of these results and to whom I am indebted for Table III.

The work was done while holding the Alice Memorial Fellowship and with a grant for expenses from the Imperial Cancer Research Fund.

### REFERENCES.

BROWNING, H. C.—(1948) *J. nat. Cancer Inst.*, **8**, 173.
DUNN, T. B.—(1953) 'Physiopathology of Cancer.' New York (Paul B. Hober), p. 123.
FEKETE, E., LITTLE, C. C. AND RICHARDSON, F. L.—(1952) *Cancer Res.*, **12**, 219.
FOULDS, L.—(1949) *Brit. J. Cancer*, **3**, 345.
HUSEBY, R. A. AND BITTNER, J. J.—(1946) *Cancer Res.*, **6**, 240.
MÜHLBOCK, O. AND VAN RIJSSEL, T. G.—(1954) *J. nat. Cancer Inst.*, **15**, 73.
MÜHLBOCK, O., VAN EBBENHORST TENBERGEN, W. AND VAN RIJSSEL, T. G.—(1952) *Ibid.*, **13**, 505.
PREHN, R. T., MAIN, J. M. AND SCHNEIDERMAN, M.—(1954) *Ibid.*, **14**, 895.
PULLINGER, B. D.—(1947) *Brit. J. Cancer*, **1**, 177.—(1949) *Ibid.*, **3**, 494.—(1952*a*) *Ibid.*, **6**, 69.—(1952*b*) *Ibid.*, **6**, 78.—(1954) *Ibid.*, **8**, 161.